# Monitoring and early warning method for a rockfall along railways based on vibration signal characteristics

**DOI:** 10.1038/s41598-019-43146-1

**Published:** 2019-04-29

**Authors:** Yan Yan, Ting Li, Jie Liu, Wubin Wang, Qian Su

**Affiliations:** 1 Key Laboratory of High-Speed Railway Engineering, Ministry of Education, Southwest Jiaotong University, Chengdu, 610031 China; 2 School of Civil Engineering, Southwest Jiaotong University, Chengdu, 610031 China; 3 National Engineering Laboratory for Technology of Geological Disaster Prevention in Land Transportation, Southwest Jiaotong University, Chengdu, 610031 China

**Keywords:** Natural hazards, Geomorphology

## Abstract

Rockfall disasters occur frequently in mountainous areas of western China, and the rockfall disasters along a railway line will seriously affect the safety and normal operation of railways, causing great economic and property losses. Existing rockfall monitoring and early warning methods still have shortcomings, such as accurate warning of single-point disasters and vulnerability to the natural environment. In this study, a rockfall test of a flexible safety protection net along the slope of a railway and a rockfall test of the railway track were carried out, and the vibration signals of the falling rock hitting the different sites of the protective net and hitting different positions of the rails were obtained. Using the signal analysis methods such as Fast Fourier Transformation and Short-Time Fourier Transform, the basic characteristics of the rockfall vibration signal and the vibration signal when the train passes and the propagation law of the rockfall vibration signal are obtained. Finally, a set of monitoring and early warning systems for rockfall disasters along the railway based on the analysis of vibration signal characteristics is established. The monitoring and early warning method has the advantages of all-weather, high-time, semi-automatic and high efficiency performance.

## Introduction

The mountainous terrain in China’s western regions is undulating and has complex conditions. Rockfall hazards^1^ have become one of the most serious problems jeopardizing the safety of railway lines in the western mountainous regions. According to incomplete statistics, there were 238 rockfalls at 214 sites responsible for 910 hours of 16-minute traffic interruptions in the northern section of the Chengdu-Kunming Railway from 1971 to 1992. The occurrence of rockfall hazards along the railway will seriously affect the safety and normal operation of the railway and result in great economic and property losses^2–5^. The monitoring and early warning of rockfall hazards along the railway in mountainous areas are therefore necessary.

Commonly used technologies of rockfall monitoring include light detection and ranging (LiDAR), laser scanning, geographical information systems (GISs) and video image recognition. As an information data collection method applied to the natural physical object space^6,7^, LiDAR is widely used in the monitoring, detection and evaluation of rockfall and the analysis of rockfall vibration characteristics^4,8,9^. However, owing to scattering of the scanning light, the spatial information captured by the LiDAR sensor is not yet comprehensive enough, and laser scanning is more effective^10^. Laser scanning has also been used for the monitoring of rockfall over large-scale areas^11^. In addition, researchers usually combine LiDAR, laser scanning, and GIS technology to establish a three-dimensional numerical model of a rockfall and to analyze the spatiotemporal characteristics of rockfall hazards^12,13^ and make rockfall hazard risk assessments^14^. In addition, video image recognition methods are often applied to the monitoring of rockfall hazards. Such methods can be employed to monitor the status of dangerous rocks remotely and greatly reduce the use of human resources^15,16^. Although these commonly used rockfall monitoring methods have been effective to a certain level, they still suffer deficiencies. LiDAR and laser scanning methods, for example, are suitable for the assessment of the risk of rockfall in a large area but not for the accurate monitoring and early warning of a single rockfall. Video image recognition is susceptible to factors of the natural environment, such as poor performance at night, in heavy fog and under extreme rainfall conditions. A GIS is usually used in numerical simulation for the analysis and assessment of the risk of rockfall, but its timeliness is poor.

Currently, the development of electronic technology allows the analysis of the rockfall vibration characteristics through signal monitoring. More and more research has started to monitor and provide an early warning of rockfall hazards according to the characteristics of vibration signals. Signals of the acceleration and velocity of ground vibration caused by rockfall can be collected by sensors such as geophones and accelerometers, for example. Time-frequency analysis of the vibration signals that are obtained can provide the scale and dynamic process of the rockfall, providing data for monitoring and early warnings, and such analysis is feasible and mature^17,18^. First, many studies involving signal monitoring and analysis are focused on the frequency distribution of the ground vibration generated by rockfall. The frequency distribution of ground vibration signals triggered by rockfall is related to various factors such as the scale of the rockfall and the range monitored by sensors. Field monitoring results show that the main frequency of the ground vibration signal generated by rockfall is concentrated within 10–20 Hz^19–21^, while the vibration signal frequency is lower for larger rocks^22^. Second, by analyzing the characteristics of the rockfall vibration signal, we can obtain the relationship between the physical parameters of the vibration signal and parameters such as the rockfall distance and scale. The signal duration t_30_, for example, is roughly related to the falling rock energy and rockfall distance^23^. The volume, scale and signal detection range have a linear relationship^24^. In addition, through time–frequency analysis of the rockfall vibration signal, the inverse of the rockfall hazard induction mechanism can also be obtained^25,26^. In summary, current research on the characteristics and related mechanisms of rockfall based on vibration signals is relatively complete, but there are still relatively few studies involving vibration signal analysis of rockfall disasters along a railway. D.S. Collins acquired the characteristics of a vibration signal of rockfall along a railway in North America by laying microsensors along the railway^2^ but only conducted a preliminary analysis of the time-domain characteristics of the vibration signal. In practical applications, a safety netting system is usually installed in areas of rockfall hazard along a railway. It is therefore necessary to further analyze the characteristics of vibration signals of falling rock hitting the protective net to determine the status of the net. After rockfall breaks through a protective net, it threatens the track structure below. Falling rocks hitting the track and a passing train both produce high-energy vibrational signals. To avoid a false alarm of a rockfall event due to the vibration signal of the train, it is necessary to distinguish the characteristics of the two vibration signals. In addition, based on the vibration signal characteristics, construction of the monitoring and early warning methods for rockfall hazards along a railway needs further study.

The present study carried out a rockfall test of a safety netting system and a track rockfall test to obtain the vibration signal characteristics of railway infrastructure hit by rockfall and employed the fast Fourier transform (FFT) and short-time Fourier transform (STFT) to measure the time–frequency characteristics of the rockfall vibration signals of a protective net and train-induced vibration. This paper analyzes and discusses the time–frequency characteristics of rockfall vibration signals. A monitoring and early warning method is proposed for rockfall hazards along railways according to the analysis of vibration signal characteristics, providing guidance for the prevention and treatment of rockfall hazards along railways.

## Results

### Vibration characteristics in the protective net

**Figure 1:**
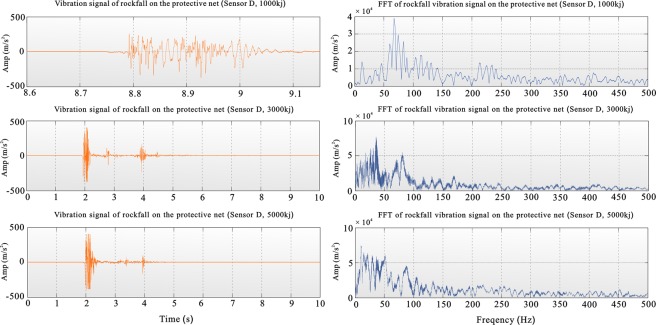
Vibration curve and frequency chart for the experimental sensor at point D on the protective net ((top) 1000-kJ drop breaking the net, (middle) 3000-kJ drop without breaking the net, and (bottom) 5000-kJ drop without breaking the net).

For the 1000-kJ drop, the protective net broke. The experimental system does not allow the heavy object to move with the protective net, and the net thus does not have a continuous effect on the large object. In practice, the protective net gradually stops the movement of a heavy object after a single impact. After hitting the net, the heavy object is balanced by its own weight, and the vibration amplitude is lower than the vibration amplitude when the protective net is not broken, whereas the vibration frequency is relatively high. The duration of the large vibration recorded for the 1000-kJ drop was short, approximately 0.2 s, and the energy dissipation was extremely fast. The dominant frequencies were concentrated in the range of 50–150 Hz. For the 3000- and 5000-kJ drops, the protective net was strengthened experimentally; the heavy object fell on the protective net and moved up and down with the protective net until the two reached equilibrium. The vibration was greatly affected by the weight. The amplitude of vibration of the protective net was therefore relatively large, and the duration of the larger amplitudes of vibration was short. The heavy and protective nets gradually converge in terms of vibration owing to the effect of the heavy object, and the high-frequency vibration is weaker than the low-frequency vibration because heavy objects have a large inhibitory effect on the high-frequency vibration of the protective net. For the 3000- and 5000-kJ drops, the vibration time recorded by the sensor was relatively long, and the dominant frequency band was concentrated at low frequencies. The best frequency band was 0–50 Hz, while the second-best frequency band was 75–100 Hz (Fig. 1).

#### Horizontal vibration characteristics of the signal

**Figure 2:**
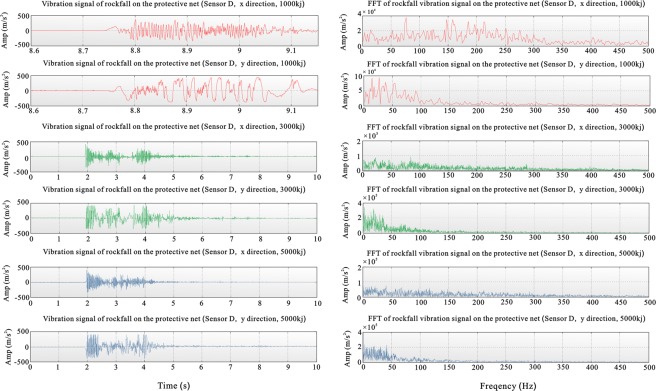
Vibration curve and frequency chart for the two horizontal sensors at point D on the protective net. (Directions x and y are horizontal and perpendicular to each other. The upper two figures show results for the 1000-kJ drop with the net breaking, the middle two figures show results for the 3000-kJ drop without breaking the net, and the lower two figures show results for the 5000-kJ drop without breaking the net).

The duration of high amplitudes of the lateral vibration was longer than the duration of high amplitudes of the vertical vibration. The horizontal vibration times for the 1000-, 3000- and 5000-kJ drops were longer than the vertical vibration times. There was a relatively large vibration amplitude in the horizontal direction (y) of the protective net experiment with respect to the x-direction, while the frequency of the horizontal vibration in the x-direction with respect to vibration in the y-direction was high. The characteristic of the frequency spectrum was that the dominant frequencies in the spectrum for horizontal direction y were concentrated in a low-frequency band, 0–50 Hz, which is the first dominant frequency band. The characteristics of the frequency spectrum and signal in the time domain, the horizontal direction and the treatment direction were similar, but the recording time in the time domain was longer and combined investigation for vertical and horizontal directions was more effective (Fig. 2).

#### Vertical vibration characteristics of the signal

**Figure 3:**
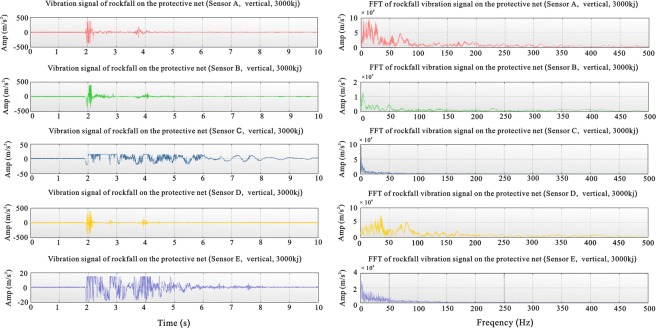
Vibration curves and frequency diagrams recorded by sensors at different positions for the 3000-kJ drop. (From top to bottom are data curves for sensors at points A, B, C, D and E, with 50 G range sensors at points A, B, and D and 2 G range sensors at points C and E).

The signal recorded by a 40 G sensor had a relatively short duration of strong amplitude and was accompanied by a small amplitude with a long duration, while the signal recorded by a 2 G range sensor had a relatively long duration. This is mainly because the 2 G sensor records mainly small vibrations, and the maximum value during the mapping was also 2 G; however, the maximum range recorded by the 40 G-range sensor was relatively large, the maximum value during the mapping was 40 G, and such low amplitudes appeared smaller in the amplitude curve. Furthermore, the sensitivities of the 40 G and 2 G sensors were different, and the 2 G sensor had a stronger recognition energy than the 40 G sensor for a weak signal. The signals recorded by the sensors at positions A, B and D were similar in the time and frequency domains. The duration of the large vibration amplitude was short while the duration of the small vibration amplitude was long. The first dominant frequency segment was 0–50 Hz, while the second dominant frequency segment was 75–100 Hz (Fig. 3).

### Vibration characteristics of the track rockfall test

#### Characteristics of the train vibration signal and rockfall signal

**Figure 4:**
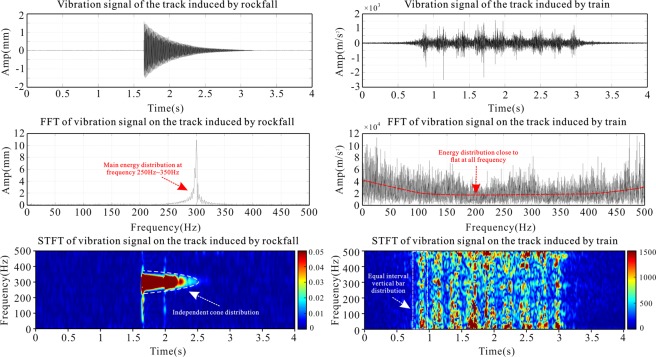
Comparison of rockfall (left) and train (right) signals in track tests.

The train signal is spindle-shaped, with the first and last spindles being unimodal and the middle spindle being bimodal. The FFT is used to transform the vibration signal of the train and rockfall to the frequency domain. The train signal has a strong energy at each frequency. The frequency characteristics of the train signal are like the frequency characteristics of the white-noise signal. There is no obvious dominant frequency band for a typical broadband vibration signal. The time-frequency spectrum obtained by STFT is in the form of a distinct vertical strip, and the spectrum also shows the characteristic of energy in the whole frequency band (Fig. 4). When the train wheels pass, the vibration amplitude of the rail is not obvious, and the rail vibration is generated mainly when the train’s bogie passes. When each bogie of the train approaches a sensor, the vibration amplitude of the rail at the sensor gradually increases. When the bogie reaches the sensor test position, the vibration amplitude reaches a maximum, the vibration amplitude of the rail gradually decreases away from the sensor test position, and the sensor records a vibration curve that has a spindle shape in the time domain. There are two pairs of bogies at the two ends of the CRH train, so that there are two pairs of bogies at the link of the car. The pair of bogies correspond to one spindle. Because the two pairs of bogies at the link of the car are very close together, the two are heavy. Paste shows the characteristics of the bimodal spindle. The signal has energy in all frequency bands, and both the spectrum and the time spectrum are reflected. Each vertical bar represents the vibration generated by the bogie of the train compartment and has energy throughout the Nyquist frequency range. However, the frequency characteristics of each vertical bar are different, indicating that the weight and movement status of each car of the train may differ.

The rockfall vibration signal has a cone shape in the time domain, and the amplitude decreases rapidly with time. The duration of the signal is approximately 1 s shorter. The spectrum of the orbital rockfall vibration signal shows a single peak, and the dominant frequency band is obvious, reaching a maximum near 300 Hz; the energy in other frequency ranges is very low. The rockfall vibration signal has a sharply tapered distribution on the time–frequency chart, and the frequency range of the rockfall signal can be clearly obtained from 250 to 350 Hz (Fig. 4). The apparent single-peak spectrum shows that the excitation source of the rockfall vibration signal is relatively simple. Both the time spectrum and the time domain curve are spindle-shaped, reflecting the fact that the signal exhibits a decaying tendency after the frequency and energy hit without a continuous secondary vibration. The time-frequency diagram has two features that overlap each other in the shape of a cone, which may be that the rock falls on the track. When the falling rock falls again after rebounding in the orbit, since the energy has been greatly reduced, the phenomenon that the two cones overlap each other in the time domain is not obvious; and in the frequency domain, the frequency of the signal due to the two drops is generated. The range is very similar, so the two spectral cones appear to be more clearly overlapping in the time spectrum.

#### Vibration characteristics in the track rockfall test

Signal characteristics of different hit positions: The length of the signal was approximately 1 s, but the duration of each experimental signal was not the same; i.e., the time-domain morphologies of the signals were largely similar, both being cone-shaped, but the amplitude was different for each signal. The power generated by falling rock hitting the edge of a sleeper near sensors was higher than the power generated by falling rock hitting the center of a sleeper. The energy of an impact on the rail buried with sensors was greater than the energy of an impact on the center of a sleeper. The energy decreased in the order of edge of a sleeper near sensors > center of a sleeper > rail buried with sensors > rail away from sensors. The energy quickly attenuated.

The spectral characteristics of the four curves are similar in that the frequency is extremely high, the frequency bandwidth of the frequency spectrum is narrow, the main frequency is approximately 300 Hz, and the main frequency shifts slightly owing to the different impact positions and impact energies. The reason for the approximately single-frequency component may be that there is only one strike of a fixed-mass rock, having a frequency of vibration mainly approximately 300 Hz, and there is no rockfall from different heights generating signals with other main frequencies at the same time.

**Figure 5:**
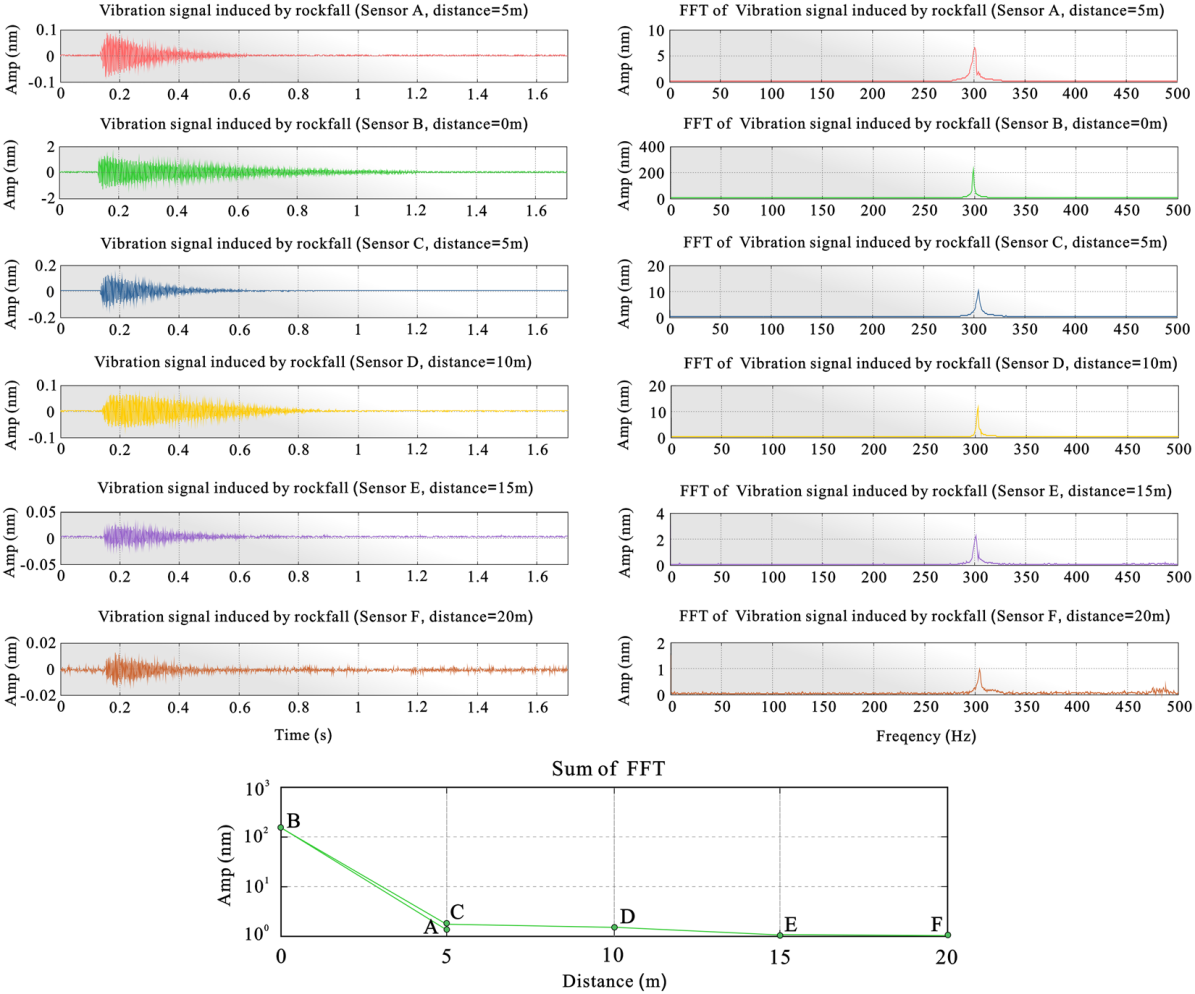
Signal characteristics of rockfall striking the edge of a sleeper near the sensors at different positions ((left) time-domain characteristics of the signal, (middle) FFT spectrum, (right) signal energy changes recorded by sensors at different distances from the impact point).

Signal propagation characteristics analysis: The signals recorded by the sensors in different positions have great similarities in both time and frequency domains. All time-domain signals are cone-shaped. The main frequency of the frequency domain is approximately 300 Hz, but the signal energy increases with the propagation distance. However, the signal energy rapidly decreases with an increase in the propagation distance (Fig. 5), and the energy of the signal decreases rapidly. In the process of signal transmission on the track, the frequency characteristics and curve characteristics of the signal do not change greatly, but the signal energy attenuates quickly, mainly because the frequency of the signal is extremely high. During signal propagation, the high-frequency signal components are in the process of transmission. The high-frequency signal components transmit much more rapidly than the lower-frequency components.

### Railway rockfall monitoring and early warning system

#### Design criteria for monitoring system of falling rock protection net

Combined monitoring with vertical and horizontal sensors having different precision: When laying out the sensors, it is not necessary to place sensors at different positions on the protective net or to perform experiments on the placement positions. The sensors are placed in a convenient manner. At the same time, when the horizontal sensor is placed, the direction of the maximum horizontal vibration needs to be tested to find the direction in which the vibration in the horizontal direction is the greatest, and an acceleration sensor in the horizontal direction is arranged along this direction and the vertical direction. Vertical vibration must be recorded with sensors having two different ranges. Low-range, high-sensitivity sensors are used to record environmental noise and to monitor small rocks for slippage. Large-scale sensors are used to monitor the falling of large rocks and can be used horizontally.

Judging whether the protective net is punctured according to characteristics of the vibration signal: The above analysis reveals a substantial difference between the signal recorded by the sensor and the signal recorded when the protective net is broken down. First, the scenario can be distinguished from the duration of vibration in that the vibration signal lasts longer than 2 s when the rail is not hit by a falling rock. When the net is punctured, the recorded vibration signal has a short duration of approximately 0.2 s. Again, it can be judged from the forms of curves in the time and frequency domains that the amplitude of the recorded signal is relatively small when the net is punctured, and it is slow when the change in the frequency of the curve is relatively punctured. Finally, the main frequency range of the recorded signal can be used for judgment, with a curve having a higher main frequency being more likely to correspond to a break in the net.

#### Design criteria for track rockfall monitoring subsystem

Rockfall signal recognition method: Time-domain analysis of the vibration signal is first conducted to differentiate the train signal and the rockfall signal. The train signal has a spindle shape, and the number of spindles is related to the number of bogies of the train. The signal of the falling rock striking the track is tapered. If the rock falls on the track, there may be two conical end-to-end encounters, but the phenomenon is not obvious in most cases. The FFT is applied to the signal to obtain the frequency spectrum of the signal. The single-peak frequency spectrum is that of a rockfall vibration signal. The main frequency of the peak spectrum is determined by the scale of the rockfall and increases with the energy of the falling rock. The primary frequency is likely to increase as the power of the rockfall increases. When the frequency spectrum shows irregular white noise, the vibration signal is that of a train. When the STFT is applied to the signal, the time spectrum of the train signal appears to have a strip shape. The signal of falling rock has a relatively clear feature of two conical end-to-end connections.

How to determine the position of rockfall: The energy of the signal generated when rock falls on the track attenuates quickly as the signal travels along the track. The position of the rockfall can therefore be determined by the position of impact and the position of a sensor. Meanwhile, the sensor needs to be placed in or near the hidden danger zone of the rockfall. Otherwise, owing to the rapid attenuation of the signal energy during signal propagation, the sensor cannot record the signal generated by the falling rock striking the track. At the same time, the signal features of the sensors on the track can be used to analyze the approximate location of the rockfall. We can find the impact point of the falling rock according to the relationship of the energy curve of the vibration signal to facilitate quick troubleshooting.

#### Rockfall monitoring and early warning system

To monitor whether the falling rock penetrates the protective net and whether the rock falls on the track after penetrating the protective net, a falling-rock signal monitoring system is established. The basis of the system is to place corresponding acceleration sensors on the track and protective net to detect vibration characteristics.

Using the rockfall signal monitoring system, it is possible to monitor any vibration signal from rock that falls on the track after penetrating the protective net. If the rock falls on the protective net, does not break through the protective net and remains above the protective net, then there is only the vibration signal of the protective net, and the vibration signal on the rail is the background value. If the falling rock breaks through the protective net and falls on the track, vibration signals are recorded on the protective net and on the track, and the vibration signal of the protective net satisfies the breakdown characteristics. The vibration signal of the rail is a rockfall vibration signal or another signal. Therefore, a comprehensive analysis of the characteristics of the protective net and track vibration signals can determine the final state of the falling rock on the railway; i.e., the falling rock is blocked by the protective net, the falling rock penetrates the protective net, or the falling rock strikes the track having passed through the protective net.

## Discussion

Characteristics of the vibration signals of static, broken and unbroken protective nets were clarified, and a method of distinguishing the state of rockfall was established. Such knowledge of the state of falling rock allows the clear determination of whether rockfall is affecting railway operation. Additionally, false alarms triggered by excessive ambient noise can be eliminated.

Through analysis of vibration signals in time and frequency domains, the train signal and rockfall signal can be clearly distinguished. By installing a sensor system on the track, we can quickly determine the impact point of the falling rock. The arrangement of multiple sensors avoids the problem of the rockfall impact being far from a sensor and the vibration signal attenuating greatly along the track during transmission.

The present study is purely qualitative. It analyzes only the signal characteristics of rockfall in various situations for a protective net and track and does not discuss differences in vibration signals due to rocks being of different scales. The system cannot provide a quantitative estimate of the scale of the rockfall. At the same time, the experiments ignored the signal feature analysis next to rock striking the track, making it impossible for the system to directly identify the situation where rockfall penetrates the protective net but does not fall on the track and instead lies on the open ground next to the track. Only an indirect judgment can be made on whether the characteristics of the signal of the rockfall are consistent with rockfall penetrating the net and falling on the track. These limitations will be solved gradually in future work.

## Methods

The present study consisted of a protective netting rockfall test and railway rockfall test. The purpose of the protective netting rockfall test is to study the characteristics of the vibration signal of the protective net in the case where falling rocks do not break the net. The purpose of the railway rockfall test is to distinguish the train-induced vibration and to analyze in depth the time–frequency characteristics of the vibration signal of the rockfall signal hitting the track.

### Protective netting rockfall test

**Figure 6:**
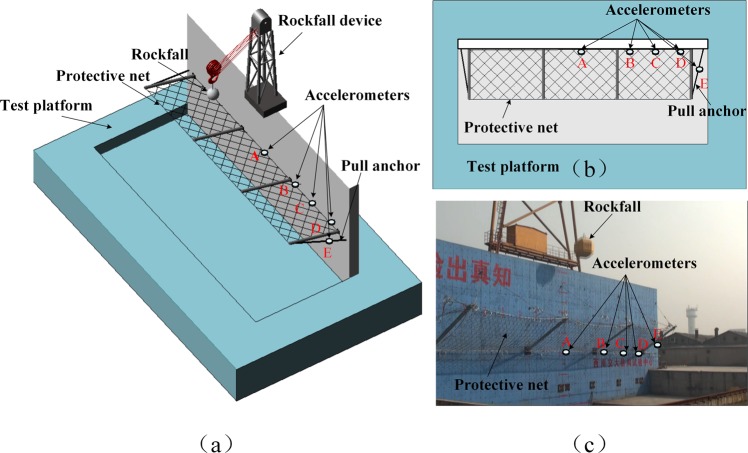
Schematic and site photographs of the rockfall protective net: **(a)** three-dimensional schematic diagram of the rockfall protection net test, **(b)** overhead schematic sketch of the experiment, and **(c)** photograph of the test site. (Measurement points A and B are the locations of vertical-acceleration sensors with a maximum recording of 40 G, points C and E are the locations of vertical-acceleration sensors with a maximum recording of 2 G, and point D is the location of two horizontal-acceleration sensors with a maximum recording of 40 G placed perpendicular to each other).

The experimental platform used in the protective netting rockfall test is the first Chinese rockfall impact test platform (Fig. 6) built by Sichuan Oster Company in 2011. This experiment was organized by Tianjin Smart Sensor Technology Co., Ltd. The safety netting is designed in strict accordance with “Railway Safety Slopes along the Railway Line” (TB/T 3089–2004)^27,28^. In the experiment, five monitoring points were set to monitor the vibration of the protective net. Four were located on the protective net and one on the pull anchor (Fig. 6a,b).

### Rockfall experiment on a railway track

#### Monitoring the signal induced by a passing train

Using an acceleration monitoring system for rails, vibration signals from the rails generated during the passing of trains in the test section of the Kunming Railway Bureau were recorded.

#### Track rockfall experiment

**Figure 7:**
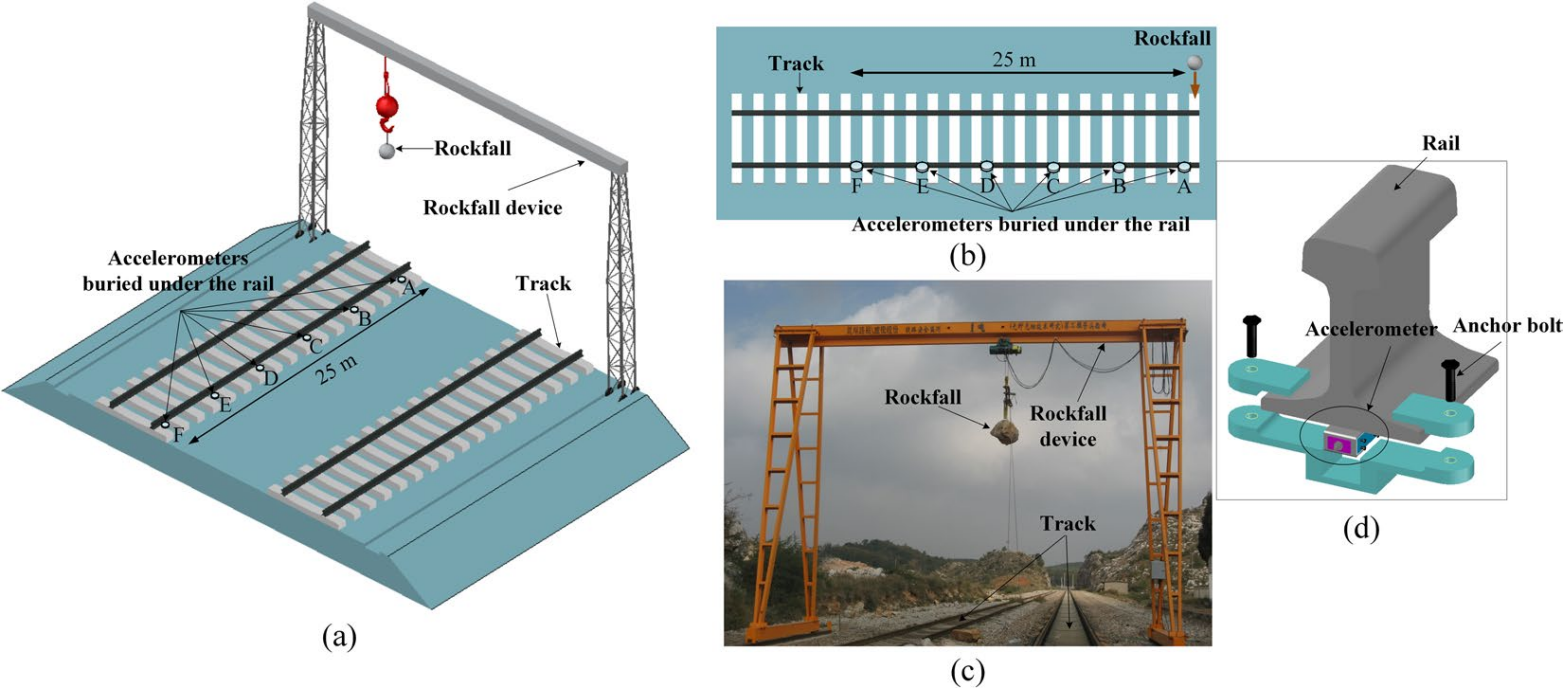
Schematic diagram and site photograph of the track rockfall experiment: **(a)** three-dimensional sketch of the rockfall impact track test, **(b)** top view of the test, **(c)** photograph of the test site, and **(d)** diagram of the sensor track installation. (The impact position refers to the part of the track struck by the rockfall: 1) the center of a sleeper, 2) the rail away from the sensors, 3) the rail buried with the sensors, and 4) the edge of a sleeper near the sensors. The impact distance is the distance between the impact point and the first detector A along the rail. FBG sensors were installed at measuring points A, B, C, D, E and F, with intervals of 5 m, to record the vibration signal).

The rockfall track test used a fiber grating (FBG) sensor system developed by Tsinghua Tongfang Co., Ltd. The system collects a rail vibration signal and generates a light wave signal. The vibration condition is obtained by changing the light wavelength. FBG sensors of the system were arranged every 5 m on the track in the section that was monitored. Each FBG sensor was installed on the lower surface of the rail, as shown in Fig. 7. An FBG sensor sampled the vibration signal every 1 ms to provide analytical data for subsequent experimental analysis. At the same time, the propagation characteristics of the vibration signal on the track could be explored. The experimental scheme is presented in Table 1.

**Table 1:** Track rockfall experimental scheme.

	Experiment 1	Experiment 2	Experiment 3	Experiment 4	Experiment 5	Experiment 6
Hit location	Center of sleeper	Center of sleeper	Edge of the sleeper near sensors	Rail buried with sensors	Rail away from sensors	Rail away from sensors
Hit distance (m)	0	5	5	0	0	5
Rockfall quality (kg)	10.4	10.4	10.4	10	10	10

### FFT

Because the vibration signal time series represents the change in signal amplitude with time, it is impossible to intuitively understand the distribution characteristics of the signal on the frequency. The FFT is introduced to transform the vibration signal from the time domain to the frequency domain. The intensities and distributions of signals in the frequency domain distinguish different sources of vibration.

### STFT

Because a single time-domain analysis or frequency-domain analysis cannot obtain the time and frequency information from a specific event at the same time, the vibration analysis of a rockfall needs to analyze not only the frequency components of the entire rockfall process but the specific moments of vibration of these frequency components. The STFT is used to perform the joint time-domain transform on the vibration signal, which not only retains the time-domain distribution information of the signal but also obtains the frequency-domain information of the signal.

### Ethical approval and informed consent

The authors of this manuscript entitled “Monitoring and early warning method for a rockfall along railways based on vibration signal characteristics” are Yan Yan,Ting Li, Jie Liu, Wubin Wang, Qian Su. This paper does not address any behavior that violates academic ethics.

### Permission document

The authors of this manuscript entitled “Monitoring and early warning method for a rockfall along railways based on vibration signal characteristics” are Yan Yan,Ting Li, Jie Liu, Wubin Wang, Qian Su. We state permission is granted to Springer Nature Limited, to publish the image under a CC BY open access license. Permission is granted to publish the image in all formats i.e. print and digital.

### Third party rights

The authors of this manuscript entitled “Monitoring and early warning method for a rockfall along railways based on vibration signal characteristics” are Yan Yan,Ting Li, Jie Liu, Wubin Wang, Qian Su. We comply with any third party rights statement in this journal.

## Data Availability

The purpose of writing this letter is to tell everyone that all the research data we use in this manuscript entitled “Monitoring and early warning method for a rockfall along railways based on vibration signal characteristics” can be made public. All the research data in this article is true and reliable. These data include the vibration signal of protective netting rockfall test, the vibration signal of rockfall experiment on a railway track, and the vibration signal induced by train. All of these data can be obtained by email from the first author Dr. Yan Yan. Yan Yan’s e-mail: yanyanyale@foxmail.com.
